# Clinicopathological Significance of the ET Axis in Human Oral Squamous Cell Carcinoma

**DOI:** 10.1007/s12253-018-0514-5

**Published:** 2018-10-31

**Authors:** Hiroki Miyazawa, Koroku Kato, Yutaka Kobayashi, Mariko Hirai, Iyo Kimura, Hiroko Kitahara, Natsuyo Noguchi, Hiroyuki Nakamura, Shuichi Kawashiri

**Affiliations:** 1 Department of Oral and Maxillofacial Surgery, Kanazawa University Graduate School of Medical Science, 13-1 Takara-machi, Kanazawa, 920-8641 Japan; 2 Department of Oral and Maxillofacial Surgery, Nanto Municipal Hospital, 938 Inami, Nanto, 932-0211 Japan

**Keywords:** Endothelin, ET-1, ET_A_R, ET-axis, Oral squamous cell carcinoma, Prognosis

## Abstract

The interaction between cancer cells and the surrounding microenvironment in malignant tumor tissue is known to be closely associated with cancer cell invasion and proliferation. Endothelin (ET) present in the microenvironment surrounding tumors has been reported to play a role in cancer cell invasion and proliferation by binding to receptors on the cell membrane of cancer cells. Here, we immunohistologically detected the expression of ET-1 and its receptor ET_A_R in oral squamous cell carcinoma (OSCC) and evaluated the association between the expression of each as well as their co-expression (ET-axis expression) and clinicopathological factors. A significant difference was observed between the invasion pattern as a parameter of cancer cell malignancy and the expressions of ET-1 and ET_A_R. The survival rates were significantly lower among the patients who were strongly positive for ET-1 and the ET_A_R-positive patients compared to negative patients. There was also a significant difference between ET-axis expression and the degree of histological differentiation and mode of invasion, and the survival rate of the positive cases was significantly lower than that of the negative cases. Our findings suggested that ET-axis assessments are important for assessing the malignancy of cancer cells and predicting the prognoses of OSCC patients.

## Introduction

For many years in Japan, malignant tumors have been the most common cause of death, and due to aging of the population the number of cancer patients in the country has been increasing [1]. Oral cancer accounts for approx. 3% of all cancers. Although this percentage is low, there are many patients with oral cancer for whom treatment is difficult, and poor outcomes despite treatment are often observed. In such a situation, drugs targeting molecules involved in cancer cell proliferation have been developed. Some of these drugs are now used clinically, but the numbers of responders to these drugs are limited, and novel treatment methods and drugs are necessary.

Many studies of factors associated with cancer cell proliferation, invasion, and metastasis have shown the close involvement of the microenvironment around the tumor, such as stroma cells and vascular endothelial cells [2–5]. The peptide hormone endothelin (ET) forms the ET axis by binding to its receptors, and it is involved in vasoconstriction and cell proliferation [6–10]. ET is composed of 21 amino acid residues. There are three types of ET (ET-1, −2, and − 3) and two types of receptor (ET_A_R and ET_B_R). Concerning the association between ET and cancer, ET is known to be involved in cancer cell proliferation, invasion, and metastasis, and the ET family has been reported to induce epithelial-mesenchymal transition (EMT) [11–16]. ET-1 binds to ET_A_R with high affinity, and high expressions of ET-1 and ET_A_R in tumor tissue have been reported in patients with highly malignant prostatic cancer accompanied by bone metastasis [11, 12].

Among patients with oral squamous cell carcinoma (OSCC), high ET-1 expression has been reported in OSCC patients with a poorly differentiated tumor accompanied by lymph node metastasis. However, there have been no studies on the importance of the expression of the ET axis (i.e., the co-expression of ET-1 and its receptor), and this axis is still poorly understood. Therefore, to evaluate the importance of ET-1 and ET_A_R expressions and their co-expression, we evaluated the expressions of ET-1 and ET_A_R constituting the ET axis, and analyzed their association with clinicopathological factors and prognosis in patients with OSCC.

## Materials and Methods

### Tissue Samples

The subjects were 74 patients who visited the Department of Oral and Maxillofacial Surgery of our hospital between January 2000 and December 2015, underwent surgical resection or tissue biopsy, and were histopathologically diagnosed with OSCC. There were 36 males and 38 females aged 29–89 years (mean, 63.8 years). The Union for International Cancer Control (UICC) system (ver. 7) [17] was used for the TNM classification and the clinical stage; the classification by the World Health Organization (WHO) are used for the degree of differentiation, and Yamamoto’s classification [18] was used for the cancer invasion pattern.

### Immunohistochemistry

To confirm ET-1 and ET_A_R expressions in tissue samples from OSCC patients, we performed immunohistochemical staining. Each tissue sample was fixed in 10% buffered formalin (Sigma-Aldrich Japan, Tokyo), embedded in paraffin, and cut into serial sections (approx. 4 μm). After deparaffinization and antigen activation, immunostaining was performed using the catalyzed signal amplification (CSA) method. As the primary antibodies, Anti-Endothelin 1 antibody (Abcam, Cambridge, MA) and Anti-Endothelin A Receptor antibody (Abcam) diluted 500-fold in phosphate-buffered saline (PBS) were used. To evaluate the specificity of the staining, we used Universal Negative Control for IS-Series Rabbit Primary Antibodies (Dako Japan, Tokyo) as a negative control. For chromogenic detection, 3, 3′-diaminobenzidene tetrahydrochloride (DAB) was used. As a counterstain, Mayer’s hematoxylin was used.

### Evaluation of Staining

Staining was evaluated based on the number of stained tumor cells using a modification of Kato’s method [19]. ET-1 and ET_A_R expressions were evaluated by an immunoassay and were examined for three fields of invasive front per specimens by microscopy at 100× magnification. The percentage of positive cells was calculated as the proportion of positive cells among 500 cancer cells in each field. For both ET-1 and ET_A_R, the threshold for positive cases was set at 50% close to the median; cases with a positive cell proportion < 50% were categorized as weakly positive cases, and the ≥50% cases were considered strongly positive cases. A case that was not stained at all was counted as negative.

### Statistical Analysis

For the statistical analysis, JMP13.0 software (SAS Institute Inc., Cary, NC, USA) was used. Differences between pairs of groups were analyzed using the χ^2^ test, and *p* < 0.05 was regarded as significant. The 5-year survival rates in the two groups were analyzed using the Kaplan-Meier method, and a multivariate analysis was performed using the Cox proportional hazard regression model.

## Results

**Fig. 1:**
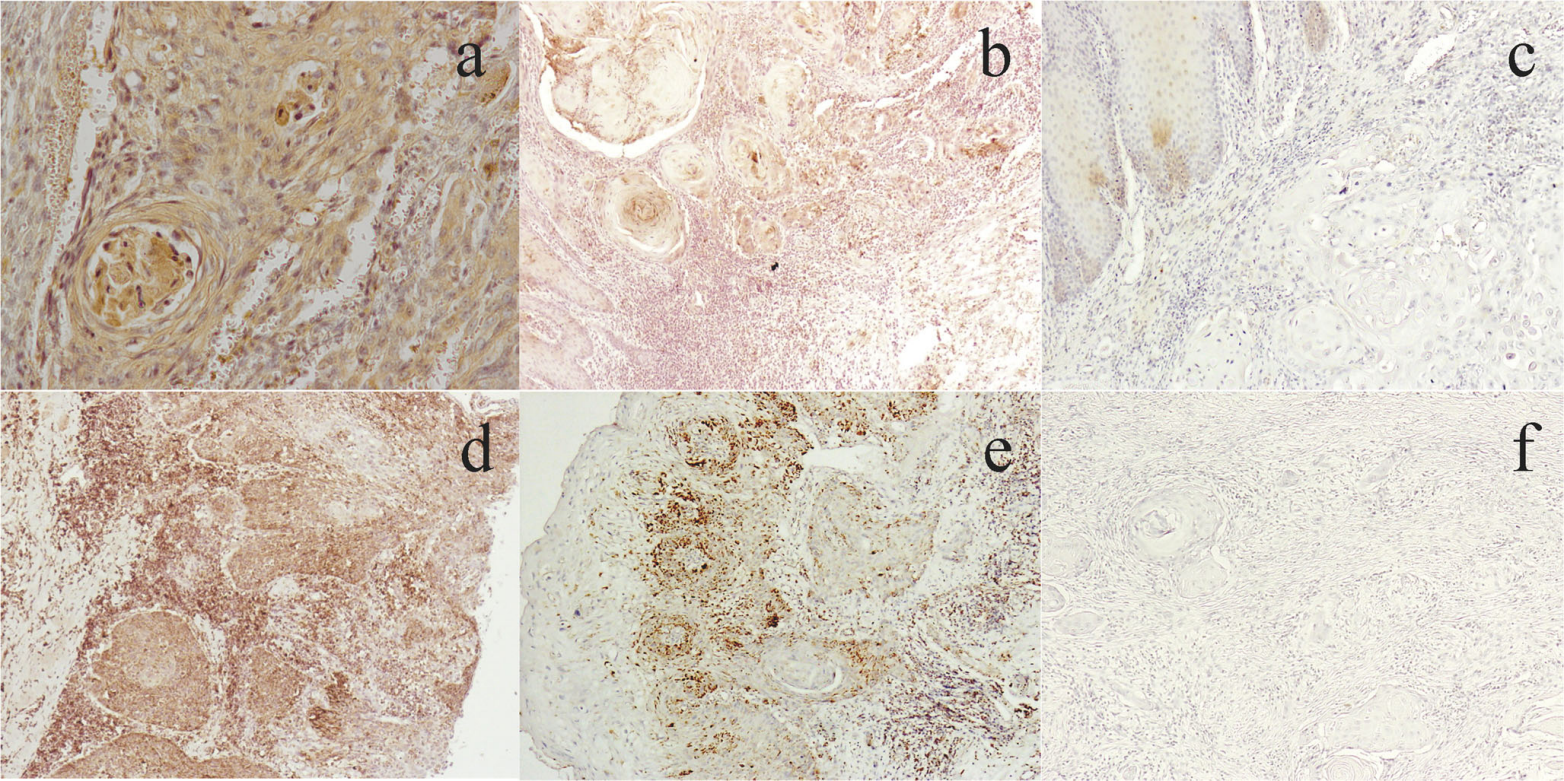
Immunohistochemical staining of ET-1 (**a**: strongly positive, **b**: weakly positive, **c**: negative) and ET_A_R (d: strongly positive, **e**: weakly positive, **f**: negative) in OSCC. ET-1 immunoreactivity is observed in the cytoplasm and cell membrane of the tumor cells, and ET_A_R immunoreactivity is observed in the cell membrane of the tumor cells (original magnification ×100)

High levels of ET-1 expression were observed in the cytoplasm and cell membrane of the tumor cells, and high levels of ET_A_R expression were observed in the cell membrane of the tumor cells (Fig. 1). Patients positive for both ET-1 and ET_A_R showed their expressions in not only tumor cells but also stroma cells including fibroblasts, and all cases that showed expression in stroma cells also showed expression in tumor cells.

Table 1 summarizes the relationship between clinicopathological factors and the expressions of ET-1 and ET_A_R. Among the 74 OSCC patients, there were 43 patients who were strongly positive for ET-1 (58.1%), 16 patients who were weakly positive for ET-1 (21.6%). Thirty-seven of the patients were strongly positive for ET_A_R (47.3%), and 19 patients were weakly positive for ET_A_R (25.7%).

**Table 1:** Clinicopathological parameters in relation to ET-1 and ET_A_R expression (*n* = 74)

Variables	*n*	ET-1 no. (%)			*P* value	ET _A_R no. (%)			*P* value
		Strongly	Weakly	Negative		Strongly	Weakly	Negative	
Age, years					0.28				0.69
< 65	35	17 (48.6)	9 (25.7)	9 (25.7)		16 (45.7)	9 (25.7)	10 (28.6)	
≥ 65	39	26 (66.7)	7 (17.9)	6 (15.4)		21 (53.9)	10 (25.6)	8 (20.5)	
Gender					0.27				0.26
Male	36	21 (58.3)	10 (27.8)	5 (13.9)		21 (58.3)	9 (25.0)	6 (16.7)	
Female	38	22 (57.9)	6 (15.8)	10 (26.3)		16 (42.1)	10 (26.3)	12 (31.6)	
Primary sites					0.39				0.94
Tongue	39	19 (48.7)	13 (33.3)	7 (18.0)		21 (53.9)	10 (25.6)	8 (20.5)	
Buccal mucosa	12	9 (75.0)	0 (0.0)	3 (25.0)		6 (50.0)	3 (25.0)	3 (25.0)	
Upper gingiva	12	8 (66.7)	1 (8.3)	3 (25.0)		4 (33.4)	4 (33.3)	4 (33.3)	
Lower gingiva	10	6 (60.0)	2 (20.0)	2 (20.0)		5 (50.0)	2 (20.0)	3 (30.0)	
Others	1	1 (100.0)	0 (0.0)	0 (0.0)		1 (100.0)	0 (0.0)	0 (0.0)	
T category					0.15				0.16
T1	18	10 (55.6)	2 (11.1)	6 (33.3)		6 (33.3)	3 (16.7)	9 (50.0)	
T2	36	19 (52.8)	12 (33.3)	5 (13.9)		20 (55.5)	10 (27.8)	6 (16.7)	
T3	9	5 (55.6)	1 (11.1)	3 (33.3)		4 (44.5)	3 (33.3)	2 (22.2)	
T4	11	9 (81.8)	1 (9.1)	1 (9.1)		7 (63.6)	3 (27.3)	1 (9.1)	
N category					0.58				0.13
N (−)	58	32 (55.2)	13 (22.4)	13 (22.4)		28 (48.3)	13 (22.4)	17 (29.3)	
N (+)	16	11 (68.8)	3 (18.7)	2 (12.5)		9 (56.3)	6 (37.5)	1 (6.2)	
Stage					0.07				0.03
S1	16	9 (56.3)	1 (6.2)	6 (37.5)		6 (37.5)	1 (6.2)	9 (56.3)	
S2	31	15 (48.4)	11 (35.5)	5 (16.1)		16 (51.6)	9 (29.0)	6 (19.4)	
S3	11	6 (54.5)	2 (18.2)	3 (27.3)		5 (45.4)	4 (36.4)	2 (18.2)	
S4	16	13 (81.3)	2 (12.5)	1 (6.2)		10 (62.5)	5 (31.3)	1 (6.2)	
Cell differentiation					0.08				0.08
Well	31	14 (45.2)	9 (29.0)	8 (25.8)		15 (48.4)	6 (19.4)	10 (32.2)	
Moderate	26	15 (57.7)	4 (15.4)	7 (26.9)		10 (38.4)	8 (30.8)	8 (30.8)	
Poor	17	14 (82.4)	3 (17.6)	0 (0.0)		12 (70.6)	5 (29.4)	0 (0.0)	
Mode of invasion					0.04				<0.01
1	5	1 (20.0)	2 (40.0)	2 (40.0)		2 (40.0)	0 (0.0)	3 (60.0)	
2	11	3 (27.3)	2 (18.1)	6 (54.6)		2 (18.2)	2 (18.2)	7 (63.6)	
3	27	17 (63.0)	5 (18.5)	5 (18.5)		12 (44.5)	9 (33.3)	6 (22.2)	
4C	21	15 (71.4)	4 (19.1)	2 (9.5)		14 (66.7)	5 (23.8)	2 (9.5)	
4D	10	7 (70.0)	3 (30.0)	0 (0.0)		7 (70.0)	3 (30.0)	0 (0.0)	

Regarding the clinical factors, no significant difference was observed in age, gender, the primary site, T classification, or N classification between the groups based on the expressions of ET-1 and ET_A_R, and there was a significant difference only between Stage and the ET_A_R expression (*p* = 0.03). Concerning the degree of histopathological differentiation, a high proportion of cases expressing both ET1 and ET_A_R was observed in the poorly differentiated squamous cell carcinomas, but no significant difference was found between the differentiation degree and the ET-1 and ET_A_R expressions. Regarding the mode of invasion pattern, as the invasion progressed, the proportion of cases in which ET-1 and ET_A_R were strongly or weakly expressed increased, and instead the percentage of negative cases decreased. A significant difference was observed between the mode of invasion and the expression of ET-1 (*p* = 0.04) and the expression of ET_A_R (*p* < 0.01).

**Fig. 2:**
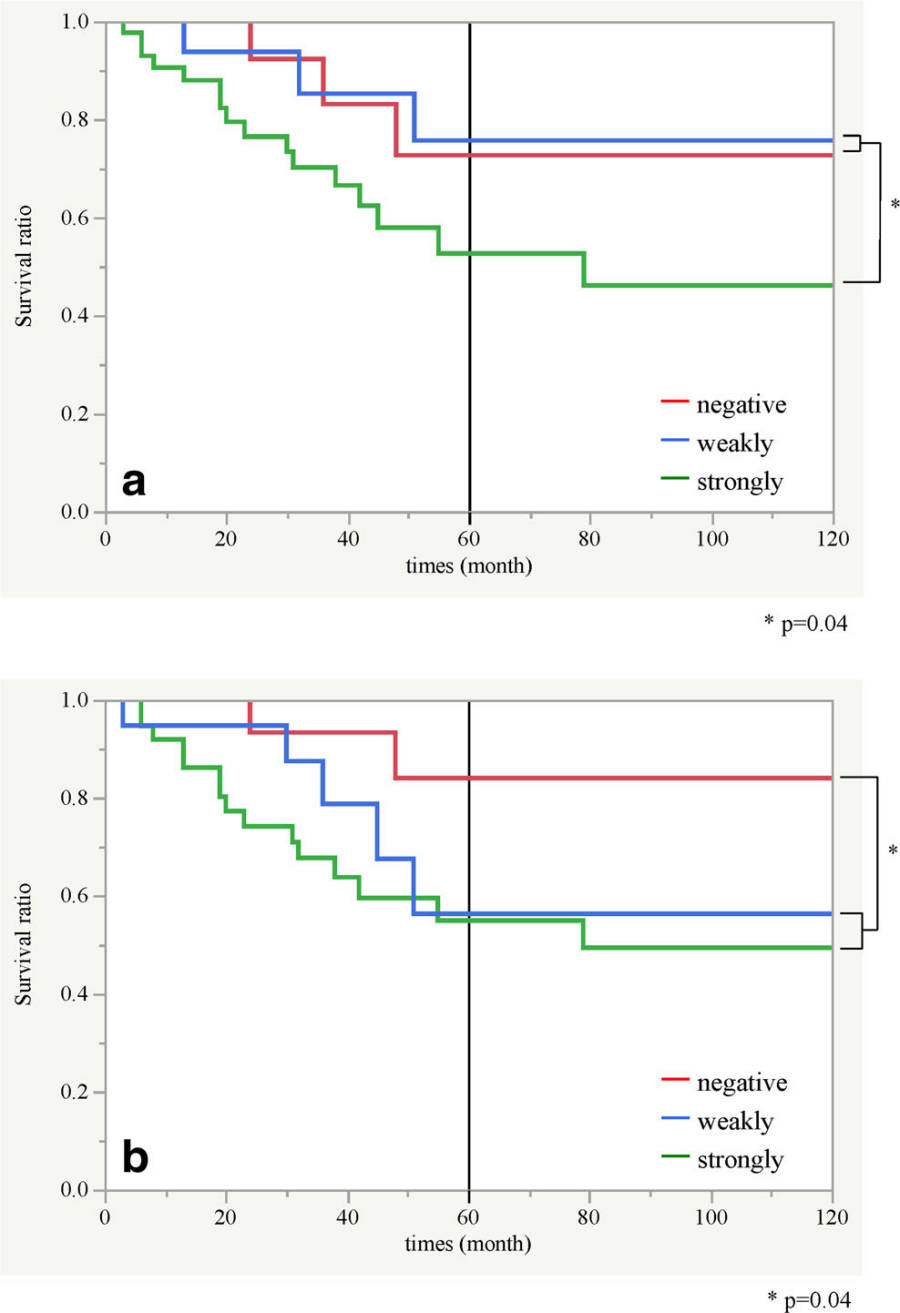
Kaplan-Meier survival estimates for overall survival based on ET-1 (**a**) and ET_A_R (**b**) expression

We evaluated the relationship between the 5-year cumulative survival rate and ET-1 or ET_A_R expression. The 5-year cumulative survival rate of the patients who were strongly positive for ET-1 was 52.7%, whereas the 5-year cumulative survival rates of negative and weakly positive patients were 72.7% and 75.8%, respectively. The survival rate was significantly lower in the ET-1-positive patients than in the negative and weakly positive patients (*p* = 0.04, Fig. 2a). In addition, the 5-year cumulative survival rates of the patients who were strongly positive and weakly positive for ET_A_R were 54.9% and 56.2%, respectively, and the 5-year cumulative survival rate of the negative patients was 84.0%. The survival rate was significantly lower in patients who were strongly or weakly positive for ET_A_R compared to the negative patients (*p* = 0.04, Fig. 2b).

**Fig. 3:**
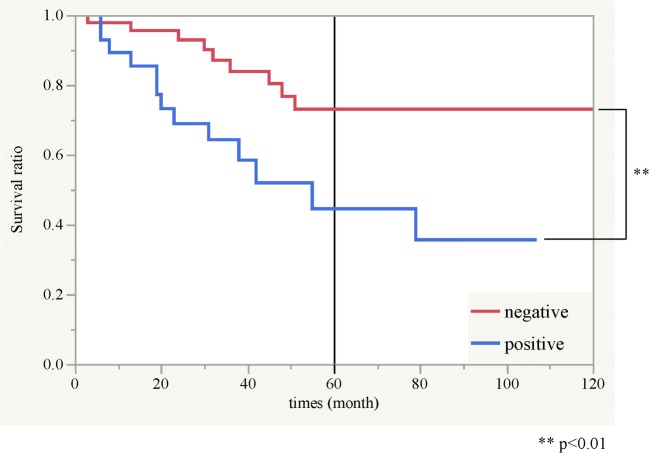
Kaplan-Meier survival estimates for overall survival based on ET-axis expression

Based on the above results, we defined the cases in which ET-1 was strongly expressed and ET_A_R was weakly and strongly expressed as ET-axis positive cases, and we defined the cases showing other expression patterns as negative cases. We examined the relationship between ET-axis expression and clinicopathological factors (Table 2). There was no significant difference in clinical factors, but there was a significant difference in histopathological factors. As the degree of differentiation decreased, and as the mode of invasion became higher, the proportion of ET-axis positive cases increased (*p* < 0.01, both factors). We next examined the relationship between ET-axis expression and the survival rate. The 5-year cumulative survival rate of the ET-axis-positive cases was 44.5%, whereas that of the negative case was 73.1%. There was a significant difference between the positive cases and negative cases (*p* < 0.01, Fig. 3).

**Table 2:** Clinicopathological parameters in relation to ET-axis expression (*n* = 74)

Variables	*n*	ET-axis no. (%)		*P* value
		Positive	Negative	
Age, years				0.17
< 65	35	16 (45.7)	19 (54.3)	
> 65	39	24 (61.5)	15 (38.5)	
Gender				0.83
Male	36	19 (52.8)	17 (47.2)	
Female	38	21 (55.3)	17 (44.7)	
Primary sites				0.52
Tongue	39	19 (48.7)	20 (51.3)	
Buccal mucosa	12	8 (66.7)	4 (33.3)	
Upper gingiva	12	8 (66.7)	4 (33.3)	
Lower gingiva	10	5 (50.0)	5 (50.0)	
Others	1	0 (0.0)	1 (100.0)	
T category				0.16
T1	18	7 (38.9)	11 (61.1)	
T2	36	19 (52.8)	17 (47.2)	
T3	9	5 (55.6)	4 (44.4)	
T4	11	9 (81.8)	2 (18.2)	
N category				0.18
N (−)	58	29 (50.0)	29 (50.0)	
N (+)	16	11 (68.8)	5 (31.2)	
Stage				0.07
S1	16	6 (37.5)	10 (62.5)	
S2	31	15 (48.4)	16 (51.6)	
S3	11	6 (54.5)	5 (45.5)	
S4	16	13 (81.3)	3 (18.7)	
Cell differentiation				<0.01
Well	31	11 (35.5)	20 (64.5)	
Moderate	26	15 (57.7)	11 (42.3)	
Poor	17	14 (82.4)	3 (17.6)	
Mode of invasion				<0.01
1	5	0 (0.0)	5 (100.0)	
2	11	2 (18.2)	9 (81.8)	
3	27	16 (59.3)	11 (40.7)	
4C	21	15 (71.4)	6 (28.6)	
4D	10	7 (70.0)	3 (30.0)

The results of a univariate analysis showed significant associations between the survival rate and the degree of cell differentiation, the mode of invasion, ET-1 expression, ET_A_R expression, and ET axis expression. However, a multivariate analysis showed no association between the survival rate and each of these factors, and each factor was not an independent prognostic factor (Table 3).

**Table 3:** Univariate and multivariate analyses for clinicopathological parameters, ET-1, ET_A_R, and ET-axis expression in relation to overall survival for 74 patients with OSCC

Variables	Clinical groups	Survivors (*n* = 52)	Non-survivors (*n* = 22)	Log rank		Cox regression *P* value	Risk ratio (95% CI)
				χ^2^	*P* value		
T category	T1–2 / T3–4	39/13	15/7	0.926	0.3359		
N category	N− /N+	42/10	16/6	0.731	0.3927		
Stage	S1–2 / S3–4	35/17	12/10	2.481	0.1152		
Cell differentiation	Well / mod-poor	26/26	5/17	5.953	0.0147	0.173	2.113 (0.721–6.192)
Mode of invasion	1–3 / 4C,4D	35/17	8/14	6.308	0.0120	0.215	1.812 (0.708–4.642)
ET-1	N, W / S	25/27	6/16	4.417	0.0356		
ET_A_R	N / W, S	16/36	2/20	4.124	0.0423		
ET-axis	− /+	28/24	6/16	6.635	0.0100	0.102	2.280 (0.849–6.126)

*N* Negative, *W* weakly positive*, S *strongly positive

## Discussion

In highly invasive squamous cell carcinoma, not only local progression but also a high late metastasis rate is associated with a poor prognosis. To understand the cell characteristics of OSCC and to search for prognostic factors, studies have been performed to evaluate the expressions of many genes and proteins, but prognostic factors have not yet been identified. It is thus necessary to discover novel biomarkers for OSCC.

In 1988, Yanagisawa et al. isolated a physiologically active substance (ET) with potent vasoconstriction activity from the supernatant of cultured porcine aortic endothelial cells, purified it, and determined the amino acid sequence [20]. The ET-axis consists of three types of 21 amino acid peptides, two types of rhodopsin-like G protein coupled receptor (GPCR), and endothelin-covering enzymes (ECEs). As a G protein-coupled receptor, ET_A_R is expressed on the tumor and stroma cell membranes. After binding to ET-1, signals are transmitted from the inside of the cell to the nucleus by various routes. ET-1 is thus considered to promote tumor cell proliferation and invasion due to autocrine and paracrine signaling [13].

This ET-axis activation is closely involved in the progression of various solid carcinomas, such as prostatic and ovarian carcinomas, and its mechanism has been clarified [11–16]. ET-1 has been reported to be associated with the growth and invasion of pulmonary cancer [21]. In hepatocellular carcinoma, ET_A_R activation by ET-1 regulates cancer cell invasion and migration [22]. There have been few studies on the ET-axis (consisting of ET-1 and ET_A_R) in OSCC, and the importance of its expression has not been evaluated. We therefore immunohistochemically determined the ET-1 and ET_A_R expressions in OSCC samples, and we investigated the association between their expressions and clinicopathological factors as well as prognosis.

Our evaluation of the possible association between ET-1/ET_A_R expression and clinicopathological factors revealed that ET-1 was significantly more frequently positive in patients with highly invasive carcinoma, which is similar to the results of previous studies [11, 15, 16]. Concerning ET_A_R expression, there was a significant association between ET_A_R expression and the mode of invasion, which is one of the OSCC’s pathological indicators, and this result is similar to the results of previous findings [12]. our univariate analysis revealed a significant association between ET-1 and ET_A_R expressions and the survival rate. Wülfing et al. reported that ET-1 and its receptor expressions are useful prognostic factors in breast cancer [23], and our findings is similar. Moreover, our finding may be related to the malignancy characteristics of cancer cells as described above.

The present of evaluation of the possible association between ET-axis expression and clinicopathological factors showed a significant association between ET-axis expression and the degree of histological differentiation as well as the mode of invasion. Regarding the 5-year cumulative survival rate, the prognosis of the ET-axis-positive cases was significantly poor. Correlations between ET-1 and its receptor expressions and the malignancy grade were reported in breast cancer [23], and present study’s results also suggests that the ET-axis is involved in the malignancy grade of OSCC.

In our multivariate logistic regression analysis, the expression of ET-1, ET_A_R, and ET axis were not independent prognostic factors, but our results suggest that in OSCC with high histopathological malignancy, a high mount of ET-1 is expressed and its binding to ET_A_R activates the ET-axis signal and affects the progression of OSCC. Based on the above-described results, we are convinced that an examination of the ET-axis expression in particular is important in estimating the prognoses of OSCC patients.

We evaluated the ET-1 and ET_A_R expressions in oral human squamous cell carcinoma tissue, and we observed that the evaluation of ET-axis expression as a co-expression of ET-1 and ET_A_R may be important for estimating the progression and prognosis of OSCC. A review article suggested the clinical value of ET-axis as a therapeutic target in head and neck squamous cell carcinoma (HNSCC) [24] and there is a possibility that some ET-axis-positive patients respond to the ET receptor antagonists used for the treatment of prostatic cancer [24, 25]. After further studies, the development of a novel treatment method using the ET-axis as the target is expected.
